# Analysis of Management Practices and Breeders’ Perceptions of Climate Change’s Impact to Enhance the Resilience of Sheep Production Systems: A Case Study in the Tunisian Semi-Arid Zone

**DOI:** 10.3390/ani14060885

**Published:** 2024-03-13

**Authors:** Aziza Mohamed-Brahmi, Mehrez Ameur, Ilyes Mekki, Alicia Tenza-Peral, Masarra Nasraoui, Yathreb Yagoubi, Samir Smeti, Samia Ben Saïd, Naziha Atti, Sandra Lobón, Mokhtar Mahouachi

**Affiliations:** 1 Université de Jendouba, Ecole Supérieure d’Agriculture du Kef, LR: Appui à la Durabilité des Systèmes de Production Agricoles dans la Région du Nord-Ouest, Le Kef 7119, Tunisia; ameur.mehrez@esakef.u-jendouba.tn (M.A.); bensaid.samia@esakef.u-jendouba.tn (S.B.S.); mahouachi.mokhtar@iresa.agrinet.tn (M.M.); 2 Laboratoire de Productions Animales et Fourragères, Institut National de la Recherche Agronomique, Ariana 2049, Tunisia; ilyesyassinemekki@gmail.com (I.M.); nasraoui.massara@gmail.com (M.N.); yagoubiyathreb@hotmail.fr (Y.Y.); smati.samir@inrat.ucar.tn (S.S.); naziha.belhaj@iresa.agrinet.tn (N.A.); 3 Departamento de Ciencias Agrarias y del Medio Natural, Universidad de Zaragoza, Avenida Miguel Servet 177, 50013 Zaragoza, Spain; atenza@unizar.es; 4 Instituto Agroalimentario de Aragón—IA2 (CITA-Universidad de Zaragoza), Miguel Servet 177, 50013 Zaragoza, Spain; slobon@cita-aragon.es; 5 Departamento de Ciencia Animal, Centro de Investigación y Tecnología Agroalimentaria de Aragón (CITA), Avda. Montañana 930, 50059 Zaragoza, Spain

**Keywords:** sheep, production systems, semi-arid, climate change, Tunisia

## Abstract

**Simple Summary:**

This work aims to analyze flocks’ management practices and sheep breeders’ perceptions of the impact of climate change to enhance the resilience of the sheep production systems in the Tunisian semi-arid region. The results revealed three main sheep production systems: the agro-pastoral rain-fed system (AGPRF), the agro-pastoral irrigated system (AGPI), and the agro-sylvo-pastoral system (AGSP). Each production system is characterized by specific management and productive parameters that are used by farmers to build resilience actions. However, farmers’ climate change perceptions are mostly the same across the three sheep production systems: a decrease in precipitation and an increase in temperatures and extreme events, which negatively influence feedstuff availability and costs.

**Abstract:**

Global climate change inflicts unambiguous risks on agricultural systems and food security. Small ruminants are known for their ability to adapt to changing environmental conditions. This paper aims to characterize sheep production systems in a vulnerable agricultural zone and the breeders’ perceptions of climate change to apprehend challenges that they are confronting, and formulate resilience actions. The data analysis is based on 94 semi-structured surveys of sheep farmers carried out in the Tunisian semi-arid region. The PCA analysis results revealed three main sheep production systems. The agro-pastoral rain-fed system (AGPRF) is dominant (55%), with large farms and common pastures integrating cereals and fodder. The agro-pastoral irrigated system (AGPI: 20%) is characterized by small-area and forage irrigation (1.8 ha) and a smaller number of ewes but a greater use of animal feed supplementation. The agro-sylvo-pastoral system (AGSP: 25%) is a system where grazing is based on common lands and using tree sub-products, while the agricultural area is exclusively used to cultivate cereal crops. Sheep breeders’ climate perceptions are summarized as unpredictable climate events, a decrease in precipitation, and an increase in temperature. Resilience actions principally consist of reducing flocks’ numbers, using alternative local feed, fodder, and water resources, and building more shelters and planting more trees in the grazing areas. Nevertheless, cost-effectiveness should be considered in such vulnerable zones to insure the sheep production systems’ sustainability.

## 1. Introduction

Climate change results in widely fluctuating levels of precipitation and temperature, resulting in weather instability, which in turn directly affects the agricultural sector in general and the livestock sector in particular [1]. Importantly, domestic animal species react differently to these impacts. Indeed, small ruminants are generally reared by poor farmers, particularly in developing countries, since their production systems predominantly rely on rain-fed agriculture that totally depends on natural factors [2]. Thus, small ruminants play a considerable role in securing the livelihoods of these farmers, as they have the capacity to adapt to diverse environmental circumstances due to their disease resistance, proficient grazing behavior, high feed-conversion efficiency, and drought tolerance. In addition, raising small ruminants requires low investment with maximal output, mostly because of sheep and goats’ small sizes, their prolificacy, and fewer requirements for feed and housing [3]. Generally, native sheep breeds are reared on grazing land in relatively large groups, relying on low inputs in terms of feed, water, and labor, and possess a high thermal tolerance compared to large ruminants such as cattle. They are critical for food security and livelihoods, especially under extremely stressful and diverse climatic environments [4], particularly in arid and semi-arid regions.

In Tunisia, sheep breeding is a main livestock activity. An important proportion of flocks are reared in the semi-arid and arid areas that comprise two-thirds of the country’s total area. This activity is practiced as a main agricultural, social, and sustainable resource of rural households’ livelihoods [5,6]. However, these bio-climatic regions are known for their high exposure to extreme climatic variability, such as the frequent occurrence of hot temperatures, along with a deficit of rainfall. Drought succession [7] impacts mainly wheat and other rain-fed crops that are critical to food security and livestock survival [8], which in turn threatens the agro-ecosystem in general and the livestock’s productivity, health, and sustainability in particular. In fact, during the last decade, the substantial warming tendency has become more and more pronounced during the hot season [9]. A noticeable rainfall decline is clearly affecting water resources [10] and sheep farming that is primarily based on pastures and local feed resources. In addition, climate projections for Mediterranean countries, including Tunisia, show that this area will become warmer and drier, with more frequent and extreme weather events [11,12]. Bellahirich et al. [13] presented the results of the new Tunisian projections, published in 2018 by the National Institute of Meteorology (INM). These results indicate an increase in the annual temperature forecasts for 2050 and 2100 compared to the period of 1971–2000 [14]. Specifically, average temperatures are expected to rise between 2.1 °C and 2.4 °C by 2050 and between 4.2 °C and 5.2 °C by the end of 2100. These results also predict a decrease in the annual accumulations of precipitation, varying between −1% and −14% in 2050 and between −18% and −27% in 2100 [14]. Tunisia would be subject to a more arid climate, which would affect the water and fodder resources that are intended to feed ruminant and small ruminant flocks, known especially for the adaptive capacities of the local sheep breeds. These are generally associated with cereal crop cultivation. This “cereal–fallow–sheep” system is found in 45% of farms in Tunisia, occupying 7.7% of the UAA (utilized agricultural area) [15]. Some medium to large farms have already started to diversify their activities by practicing gardening and fodder production and, sometimes, cattle breeding and/or poultry farming. On the other hand, medium and large farms are more diversified (mixed cropping–livestock), which further strengthens the autonomy of the farm, especially in terms of fertilizing their soil [16].

Generally, small-sized cereal farms own small sheep flocks and constitute the most common category of farm, particularly in the semi-arid lower climatic zone. They are the most vulnerable category, especially to the lack of fodder and/or water resources.

The objectives of this paper are to characterize the sheep production systems in the Tunisian semi-arid zone, study the households’ climate change perceptions, and propose innovative livelihood adaptation strategies to cope with these challenges, basing specific resilience actions on the identified sheep production systems in this region.

This work is included in the PRIMA ADAPT-HERD project (2019–2023): Management strategies to improve herd resilience and efficiency by harnessing the adaptive capacities of small ruminants. This project is part of the PRIMA program supported by the European Union and a consortium of Mediterranean countries (France, Spain, Tunisia, and Egypt) supported and funded under Horizon 2020, the Framework European Union’s Program for Research and Innovation.

## 2. Material and Methods

### 2.1. Study Area

**Figure 1:**
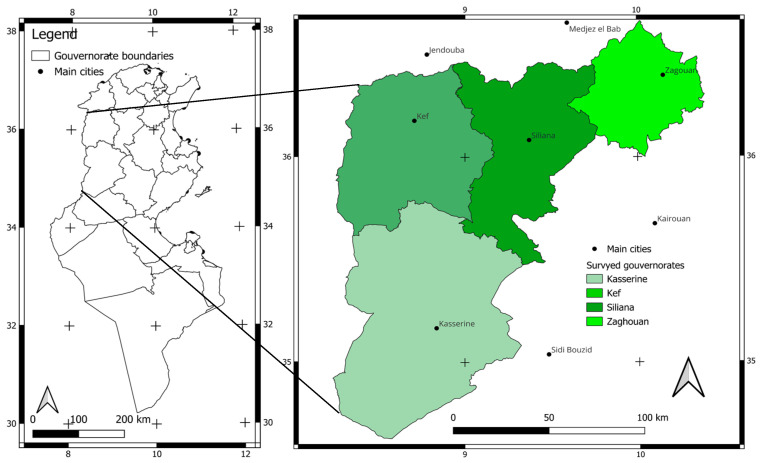
Location of the study area (present study).

The study area consisted of the Tunisian semi-arid bioclimatic regions covering northern and central parts of the country. The visited farms where this work was carried out were mainly in four governorates: Zaghouan, Kasserine, Kef, and Siliana (Figure 1). These regions represent favorable areas where farmers hold traditions of sheep breeding and lamb fattening. These governorates have an average annual precipitation of 200–400 mm/year and an annual mean temperature of 18 °C. The dry period is usually from May to August. This zone was entirely pastoral until 1960, after which it became strongly agricultural, and the farming sector has had to adapt. The arable lands represent less than 17% of the region’s surface (27,019 km^2^). Small ruminant flocks are composed mainly of sheep, with very few goats, and are concentrated in small farms averaging about 50 heads [17]. They represent 31.5% of the total sheep population in Tunisia [18]. Despite the new arable land, which had been deducted from the grazing area, the sheep population had not stopped growing. It increased from 1,377,870 ewes in 2005 to 1,442,600 ewes in 2011 [19] with an annual growth rate of 0.8%. Nevertheless, this population decreased by 0.24% (1,098,000 ewes) in 2023 [18], which could be caused mainly by limited feed resources throughout the country due to the lack of precipitation in past years.

### 2.2. Data Collection

To determine prevalent types of sheep production systems in the Tunisian semi-arid zone, a sheep farmer survey was conducted using a standardized survey that consisted of 60 questions related to the different parameters and variables linked to the farmer, the farm structure, the flock, farm management, and product marketing. In addition, 12 questions about the farmers’ perceptions of climate change were asked. The questionnaire provides homogeneous data collected from sheep farms located in the four governorate areas (Zaghouan, Siliana, Kasserine, and Kef) that were chosen after discussions with the regional animal production services of the regional OEP (Office de l’Elevage et des Pâturages) agencies in the four chosen governorates. The sampling approach was based on capturing the diversity of farm systems using open interviews with representative stakeholders. Two criteria were selected: the sheep flock size and the farm location in collaboration with the regional livestock development services and concertation with Tunisian animal sciences experts. The survey was carried out by Tunisian researchers working on the ADAPT-herd project starting with a reliability test before following the snowball sampling approach to finish with the household farms in each category. Surveys were carried out directly with sheep farmers or with one of their family members in their houses or in the grazing area. Complementary interviews were conducted with key local stakeholders that held a leadership position at the regional livestock services. Those interviews were open discussions and the interviewers filled out surveys to provide greater insight into the farming systems in the study area, their management changes over time, and the major constraints that limit their decision making. Furthermore, the objective of this work was to characterize the sheep production systems in this area, the climate perceptions by farmers, and how they adapt to these challenges.

The survey was conducted on 101 farms from 2020 to 2021. Out of the total questionnaires received, seven were incomplete or not suitable for further analysis due to contradictory or implausible information. Therefore, the sample comprised evaluable datasets from 94 farms out of 101 surveys with, globally, the same number of surveys within the four governorates. The climate perceptions are related to precipitation and the scarcity of water, the temperature increase, and the impact of climate change on forage production on sheep farms (10 questions).

### 2.3. Multivariate Analysis

A total of 94 questionnaires were subjected to statistical analysis. Principal component analysis was used for data reduction, after which the resultant nonrelated principal components (PCs) were used as inputs in the CA. The multivariate analysis approach was used in other studies to characterize production systems [20].

The PCA was carried out using 23 variables related to farm structure, management practices, and marketing criteria (Table 1).

The objective was to provide an initial approach to the variables describing the sheep production systems in the study area. Principal component analysis, or PCA, is a dimensionality reduction method that is often used to reduce the dimensionality of large datasets by transforming a large set of variables into a smaller one that still contains most of the information in the large set [21]. The PCA produced small groups of linear combinations (components or factors), which explained as much variance as possible in the original data with minimal data loss [22]. Parsimony of the principal components (PC) was verified with the Kaiser–Meyer–Olkin (KMO) test, and sample suitability was confirmed with the Bartlett test of sphericity [22]. Orthogonal varimax rotation was applied to the PCs to improve interpretation [22,23].

Linear PCA combinations were introduced into the hierarchical cluster analysis (CA) to carry out a typology of sheep production systems in the study area as a mechanism of addressing these systems’ heterogeneity [20] by grouping them into specific farm types to form groups of producers and characterize the sheep production systems. Case clustering was performed following Ward’s method, and the squared Euclidean distance was used as a measure of similarity. A dendrogram analysis and cluster coefficient were applied to identify the number of groups [23]. Because group size was not homogeneous, differences between groups were identified with an analysis of variance (quantitative variables) by comparing Hochberg means [24]. Categorical variables were analyzed with contingency tables and a χ^2^ test [23]. All statistical analyses were run with the SPSS ver. 20.0 program.

The selected 94 households were interviewed to collect their perceptions of climate change trends and impacts over the past 30 years. Most of the questions were designed as multiple-choice questions with answers in terms of one of the following: increased (I), decreased (D), no change (NC), and don’t know. The respondents who perceived an increase or decrease in climatic parameters were further asked to describe the major impacts of climate change on their sheep production systems.

## 3. Results and Discussion

### 3.1. General Characterization of the Surveyed Farms

The farmers were, on average, 45 years old, and those between 18 and 65 years were considered the active population, while unproductive labor was assigned to those younger than 15 or those older than 65 years of age [25]. As reported by Mthi et al. [26], age can affect the rate of household adoption of innovations, which, in turn, affects household productivity and livelihood strategies. They generally have a medium education level (10% illiterate, 40% primary school, 21% secondary school, 12% with professional training, 8% secondary school + professional training, and 9% high school). The farms were run mainly by family members (87%) with averages of 4.54 household size and 2 WU family labor.

Farms were, on average, 58 ha, with 0.86 sheep/UAA. The average herd was composed of 107 sheep and 5 rams. The predominant breed of ewes was the “Queue Fine de l’Ouest (QFO)” (52%). The second most common breed was the “Barbarine” (24%); crossbred or mixed sheep (Barbarine x QFO) represented 18%; and only 6% of the flocks were represented by the “Noire de Thibar” breed. Furthermore, in 75% of the surveyed farms, flocks were composed of several species (sheep, goats, and cattle). Sheep flocks grazed primarily on natural pastures and received supplementation such as commercial concentrates, barley grains, hay, straw, cactus, and olive sub-products during different periods of the year, especially before the mating period (flushing) and the lambing period (steaming). All matings were natural with a mean ratio of 1 ram: 21 ewes, with first lambing at ~15 months. The fertility and prolificacy rates were 0.88 and 1.17, respectively, with an average newborn lamb mortality of 9%. The average lamb slaughter age was ~11 months, with an average carcass weight of 15 kg.

### 3.2. Typology of the Sheep Production Systems

The typology and characterization were based on the variables presented in Table 1. The initial eigenvalues, sums of rotation of square loads, and percentage of variance explained are shown in Table 2.

**Figure 2:**
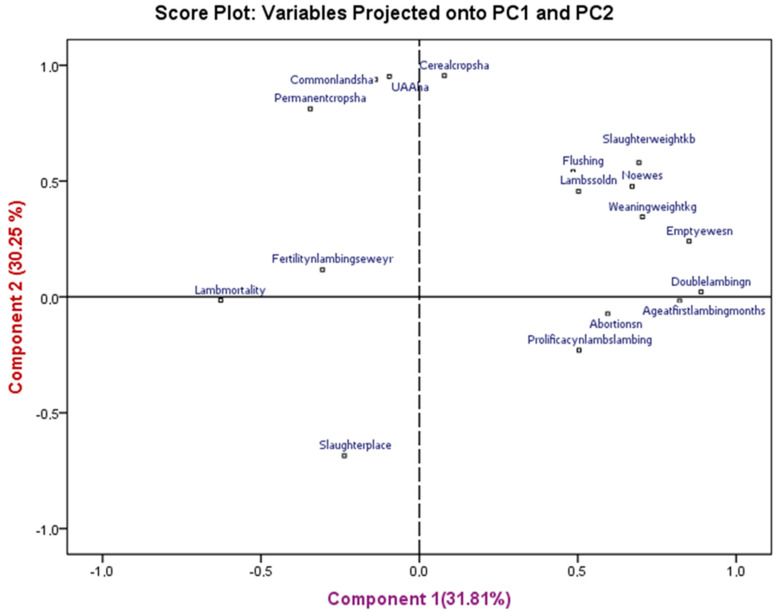
Graphic representation of modalities on axes 1 and 2.

The first two axes explained 62.06% of the total variation. The major eigenvector weights in two factors (Figure 2) were (1) fattening, commercialization, and reproductive management and (2) farm structure and crops repartition.

#### 3.2.1. Characterization of the Identified Sheep Production Systems

Three sheep production systems were obtained (Table 3): agro-pastoral rain-fed system (AGPR), agro-pastoral irrigated system AGPI, and agro-sylvo-pastoral system AGSP.

AGPRFs (55%) are characterized by large farms and common rangelands integrating cereals and fodder. AGPIs (20%) with small areas and forage irrigation (1.8 ha) had smaller numbers of ewes and greater use of animal supplementation; AGSPs (25%) were based on using common land, while the agricultural area was exclusively used to cultivate cereal crops.

##### Sheep Farmers’ Socioeconomic Characterization, Land Structure, and Use

Agro-pastoral irrigated systems had, on average, 5.9 family members, followed by agro-pastoral systems, with 4.8 members, and agro-sylvo-pastoral systems, with 3.3 members. These differences among farming systems are in line with the percentage of continuity of the farms; more family members resulted in more continuity. Sixty-one percent of the agro-sylvo-pastoral system farms did not have continuity, which indicates a future problem for the sector’s sustainability. Regarding labor, the three systems presented similar compositions of labor with a total work unit of around two per farm, being more than 90% family members for agro-pastoral farms.

The agro-pastoral rain-fed system presented the greatest UAA, with an average of 118 ha, which were dedicated to cereal crops (70 ha), permanent crops (17 ha), and forage crops (31 ha). The agro-pastoral irrigated system presented the lowest UAA, with an average near 23 ha. Thirteen were cereal crops, eight were permanent crops, and two were forage crops. The agro-sylvo-pastoral system presented an average of 59 ha of UAA, most of them cereal crops, and the rest of the crops being negligible. The UAA was mainly owned in all systems, and only the agro-pastoral irrigated system had irrigated areas, where it comprised close to half of the UAA. When the common land was considered, total land available increased, especially in the agro-sylvo-pastoral system.

The agro-sylvo-pastoral system had the highest land use, with 1.5 LU per ha UAA, whereas agro-pastoral rain-fed and agro-pastoral irrigated had 0.6 and 0.8, respectively. However, when it was expressed per ha of total land available (considering the common lands and grazing areas), the agro-pastoral irrigated had 1.3, whereas agro-pastoral rain-fed and agro-sylvo-pastoral had 0.4 and 0.6, respectively. In terms of labor, the agro-pastoral rain-fed system presented 9.3 LU per WU, agro-sylvo-pastoral system had 8.3 LU/WU, and agro-pastoral irrigated had 5.2 LU/WU.

##### Herd Management and Animal Commercialization

The agro-pastoral rain-fed system had the highest ewe number (121 ewes), followed by the agro-sylvo-pastoral system (112 ewes), with a 14% of replacement rate. The agro-pastoral irrigated system had smaller flocks, with half the ewes number (64) and a 17% replacement rate. The main breed used was QFO in both agro-pastoral rain-fed and agro-pastoral irrigated systems, and the “Barbarine” breed was the most common breed used in the agro-sylvo pastoral system.

All the surveyed farmers were not members of a breeders’ association, probably because this type of association is not well established and farmers were not yet conscious of the importance of structuring their activity. In the same line, the percentage of farmers that provided data was low (18%).

The flock source of ewes was owned or owned/bought in all systems. A low percentage of farms from both agro-pastoral systems (6–7%) bought all their replacement ewes. The source of males was similar to that of ewes, although in this case, the percentage of farms exclusively buying rams was higher, with values between 22% and 29%.

In the three systems, more than 70% of the farms were composed of mixed flocks using several species, mainly goats, followed by cattle and camels.

Agro-pastoral rain-fed sheep farmers sold their lambs by weight and by both age and weight, whereas the agro-pastoral irrigated and agro-sylvo-pastoral systems sold their lambs mainly by weight, and only 18–20% by both age and live weight. The category of lambs sold varied according to the system, with 62%, 20%, and 96% as heavy lambs (>26 kg of live weight) for agro-pastoral rain-fed, agro-pastoral irrigated, and agro-sylvo-pastoral system, respectively. The rest of the lambs were sold as light lambs.

##### Herd Reproductive Practices and Feeding Management

The main lambing systems used were continuous and one lambing/season. Some farmers used the male effect, which is a natural reproductive technique used by sheep farmers; it consists of the sudden introduction of rams to ewes that have been isolated from rams in spring, which will induce ewes to start ovulation and estrus behavior. The percentage of farms that used hormonal treatment was low, only 9% and 8% in agro-pastoral rain-fed and agro-sylvo pastoral systems, respectively. The age at first mating was similar among farming systems, between 15 and 16 months. The main lambing occurred in autumn in the three systems. The reproductive indices studied were similar among the farming systems, although the agro-sylvo-pastoral system presented some of the worst results in terms of prolificacy and productivity (Table 3).

**Figure 3:**
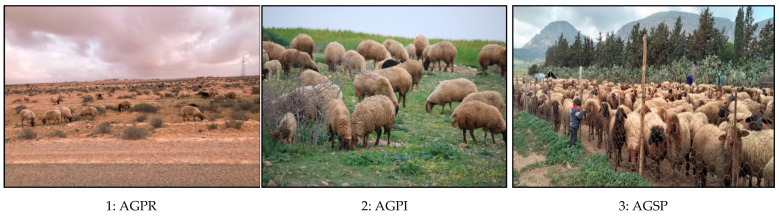
Overview of sheep production systems in the semi-arid Tunisian area: agro-pastoral rain-fed system (**1**), agro-pastoral irrigated system (**2**), and agro-sylvo-pastoral system (**3**).

The time that adult herds spend on pasture varied according to the system; in the agro-sylvo-pastoral systems, sheep flocks spent more time on pasture than the rest, averaging 330 days/year compared to 279 days/year for the agro-pastoral irrigated system and 261 days/year for the agro-pastoral rain-fed system. The type of supplement also varied according to the systems. They mainly used energy supplements or energy/fiber supplements whether indoors or outdoors (Figure 3).

In most of the surveyed farms, lambs graze with the rest of the flock (76% to 91%, according to the system). The time of grazing varied according to the system. The type of supplement depended on the period, i.e., if the lambs were suckling or not (feed concentrate or barley). More than 80% of the farms in the three systems fattened their own lambs.

### 3.3. Sheep Farmers’ Perceptions of Climatic Severity in the Study Area

#### 3.3.1. Historical and Future Climatic Conditions in the Semi-Arid Zone

Semi-arid areas are characterized by their high exposure to extreme climatic variability. In most of the Tunisian regions, the occurrence of hot temperatures along with a deficit of rainfall leads to droughts and impacts agricultural production, which will be more severe in the future. In fact, the projections of the Tunisian National Institute of Meteorology presented in Table 4 indicate a temperature increase of up to 1.8 °C by 2050 and up to 3 °C by 2100, especially in the summer and autumn seasons. Regarding precipitation, the same climate projections show a significant decrease of between 14 and 22 mm in 2050, expressing a loss of −6% and −9%, especially during winter and spring, compared to the average precipitation observed over the period of 1981–2010. The climate scenarios also predict a significant increase in climate extremes [27].

#### 3.3.2. Farmers’ Climate Perceptions: Occurrence, Manifestations, and Causes

**Figure 4:**
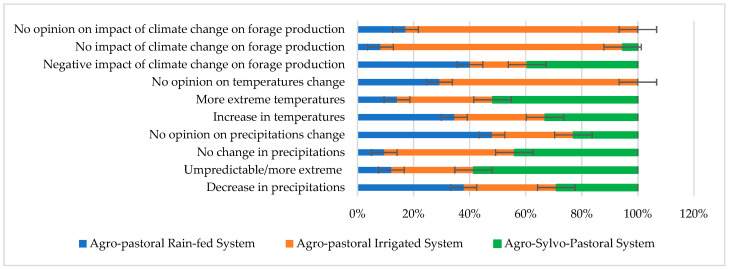
Proportions of farmers’ perceptions of general climate change in relation to the total responses by sheep production system.

Drought occurrence could be quantified by a long time series of historical indicators [7]. The sheep farmers’ climate change severity perceptions (*n* = 94) show that the surveyed farmers were generally conscious of climate change, especially the decrease in precipitation and the increase in temperature during the last decades, particularly in the cases of the agro-pastoral rain-fed and the agro-sylvo-pastoral systems. Water availability based on wells and boreholes in the case of the agro-pastoral irrigated system makes the sheep farmers more indifferent to climate change severity. Climate perceptions answers were related to precipitation and water scarcity, temperature increase, and the impact of climate change on forage production on the sheep farms. The proportions of responses for each perception in relation to the total responses are given in Figure 4.

Generally, farmers in the three sheep production systems were aware of the decrease in precipitation and the increase in temperature during the summer compared to those recorded during the last decades. In addition, most of the farmers believed that the negative impact of climate change on forage production is caused by the scarcity of water needed for crop production as a consequence of droughts, which will reduce crop yields and nutritional value and increase crop diseases and the emergence of new crop diseases, as also reported by Amamou et al. [28] in dairy cattle farms in the same areas. Perception results show that farmers in the agro-pastoral irrigated system were least concerned (47%) about the negative effect of climate change on fodder resources, whereas those in the agro-pastoral rain-fed and agro-sylvo-pastoral systems shared similar perceptions (93% and 92%, respectively).

##### Raised Climate Impacts on the Sheep Production Systems

Climate change impacts on the sheep production systems components in the study area are as follows:

- The decrease in rainfall and irrigating water;
- The decrease in pasture productivity, forage quality, and fodder resource availability;
- The variability in seasonal forage availability;
- The decrease in the availability and quality of the flocks’ drinking water;
- An increased effect of heat stress on the animals’ welfare and health;
- The decrease in individuals’ and flocks’ productive and reproductive performances; and welfare
- The increase in mortality rates because of diseases’ occurrence and emergence.

These climate impacts are summarized in Table 5.

##### General Resilience Actions

**Figure 5:**
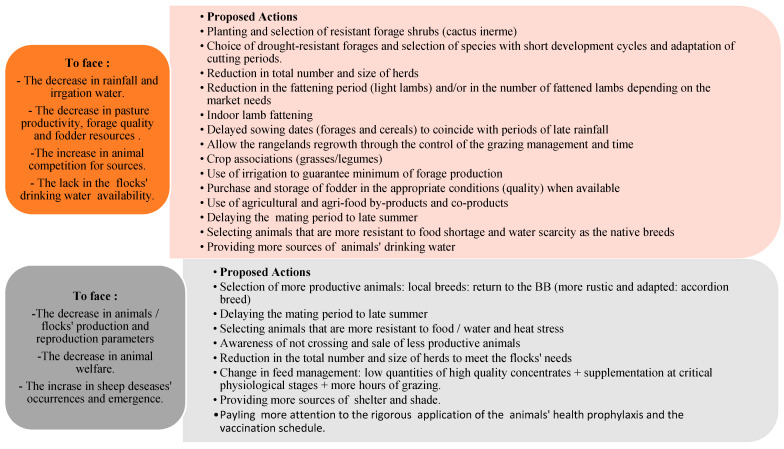
General resilience climate actions to face climate impacts on sheep-rearing activity in the study area.

Facing climate change impacts on the sheep production systems practiced in the semi-arid Tunisian zone, several actions could be proposed (Figure 5).

The proposed actions are discussed depending on the sheep production system:

- Case of the agro-pastoral irrigated sheep production system

Most of the agro-pastoral irrigated farmers focus on a feed intensification strategy as an adaptation process for the challenges and shortcomings that small ruminant breeders face, particularly in the lack of policies regulating land use and supporting agro-pastoralism, and because irrigation water availability is an asset. In fact, this Tunisian semi-arid sheep breeding case study is comparable to other rangeland ecosystems around the world, and strengthening appropriate rangeland and grazing policies that protect natural ecosystems is critical for their resilience to face the global change, as well as to enhance food security.

The feed intensification strategy focuses firstly on dairy production (cows) and secondly on sheep flocks, with the intent of producing feed resources used particularly for fattening lambs and improving reproduction and growth performances. Farmers in this case prioritize forage production by increasing forage resources at the expense of cereals or other crops. Indeed, even if cereal prices are attractive, producing their own forage allows them to have their own annual forage stocks and beat the increasing prices of concentrated feed, hay, and straw.

b. Case of the agro-pastoral rain-fed sheep production system

Sheep farmers in the agro-pastoral rain-fed system apply the same strategy as in the agro-pastoral irrigated system in wet years. Nonetheless, as the years of food shortages become increasingly frequent, fewer forage surfaces are used to intensify forage production and properties are classically used for rain fed agriculture of cereals, forage, and grazing. To restore consistency between the number of animals and forage stocks, these sheep farmers are faced with a more difficult choice between buying forage or concentrates, or reducing the number of animals to feed; although they are trying to reduce the load per hectare to allow the rangelands to regrow.

The farmers’ choice will depend on deficit extend, the type of forage lacking, the financial situation, the available forage supply, the price of concentrates, and, especially, the price of meat. Each farmer will make the decision that goes with his needs. In a context of encouraging meat prices, the first step to reducing the flocks’ number will be to anticipate culling. Further decapitalization will have to be applied once all other possibilities have been explored.

c. Case of the agro-sylvo-pastoral sheep production system

Despite being a region of agriculture and forestry, most of the area where the agro-sylvo-pastoral sheep farming system is applied has no irrigation infrastructures. The land is typically used for rain-fed agriculture of cereals and forage and grazing in addition to agroforestry.

Adapting to climate change, in this case, faces very different challenges than dealing with decreased precipitation, increased droughts, and increased temperatures. With the reduction in rainfall, forests, pastures, cereals, and forage are expected to become less productive and less economically viable. That is why, in this case, farmers focus on general farm adaptations such as the following:

- Diversification of forage systems and crops as an effective lever for securing production at every level and by using several varieties, depending on earliness, in particular;
- Use of straw, leaves, and other natural fiber to feed their flocks by creating a traditional multifunctional landscape, i.e., forestry trees combined with pastures, grazing sheep, and goats, as well as cereal or forage agriculture;
- Use of autochthone animal breeds (Barbarine and QFO).

Farmers focus on reducing the area planted with species that need a lot of water, and when it is insufficient to provide for the flocks’ feed needs, especially if all the UAA is already allocated to the herd, they would opt for a reduction in the number of livestock units to reduce the load per hectare on degraded rangelands. They also would adjust the feeding practices, purchase forage, or concentrates, limit wastage by limiting losses at the trough, and/or reduce performance targets.

## 4. Conclusions and Recommendations

For additional development and prosperity in the future, the identified sheep production systems in the Tunisian semi-arid area need to develop common and innovative approaches to meet the climate change challenge. Moreover, this research offers new views of how the present indigenous sheep breeds’ performances are challenged by climate impacts and how farmers’ practices and farm and herd management could be improved through the application of innovative tools to secure forage and water availability, ultimately guaranteeing the animals’ and flocks’ performances, health, and welfare.

## Figures and Tables

**Table 1:** Variables’ descriptions: Farm structure, management practices, and marketing criteria.

Variable Description	Abbreviation (Unit)
Utilized agricultural area	UAA (ha)
Forage crops	FC (ha)
Cereal crops	CC (ha)
Permanent crops	PC (ha)
Common lands	CL (ha)
Main breed	MB
Flushing	FL (yes/no)
Age at first lambing	AFL (months)
Productivity	PR (n lambs sold/ewe/year)
Replacement rate	RR (%)
Abortion rate	AB (%)
Empty ewes	EE (%)
Double lambing	DL (number/farm)
Lamb mortality	LM (%)
Age at weaning	AW (months)
Weaning weight	WW (kg)
Age at slaughter	AS (months)
Slaughter weight	SW (kg)
Slaughter place	SP (slaughterhouse/other places)
Carcass weight	CW (kg)
Lambs sold	LS (number of sold lambs/farm)
Selling criteria	SC (Live weight/lamb age)
Lambs for self-consumption	LFC (number of lambs for self-consumption/farm)
Continuity of the sheep-rearing activity	CN (yes/no)

**Table 2:** Total explained variance: components 1 and 2.

Total Explained Variance									
Component	Own Initial Values			Extraction Sum of Squares of Selected Factors			Sum of Squares of Rotation Factors		
	Total	% of Variance	Cumulated %	Total	% Variance	Cumulated %	Total	% Variance	Cumulated %
1	6.50	38.23	38.23	6.50	38.23	38.23	5.40	31.81	31.81
2	4.05	23.83	62.06	4.05	23.83	62.06	5.14	30.25	62.06

**Table 3:** Characteristics of the identified sheep production systems.

Variable/Production System	AGPR (55%)	AGPI (20%)	AGSP (25%)
Main breed	Queue Fine de l’Ouest (62%)	Queue Fine de l’Ouest (91%)	Barbarine (58%)
Utilized agricultural area (UAA): (ha)	118 ± 45	22.6 ± 13	59.1 ± 12
Permanent crops (ha)	16.5 ± 7	8.1 ± 4	2.3 ± 1
Common land (ha)	73 ± 39	12 ± 7	166 ± 154
Forage crops (ha)	30.9 ± 22	1.8 ± 3	0.1 ± 2
Cereal crops (ha)	70 ± 12	12.7 ± 9	56.8 ± 53
Irrigated crops (% UAA)	0	48	0
Age at first mating (months)	15 ± 5	16 ± 3	16 ± 2
Adult and lamb feed supplementation (%)	42%	67%	4%
Productivity (n lambs sold/ewe/year)	0.9	0.9	0.8
Replacement rate (%)	14	17	14
Weight as selling criteria (%)	44	80	82
Lambs mortality (%)	8	10	10
On-farm fattening (%)	87	83	88
Fertility rate (%)	90	87	85
Prolificacy rate (%)	1.19	1.23	1.10

**Table 4:** Tunisian climate projections by 2050 and 2100 [27].

	By 2050	By 2100
Temperature	+1.5 °C to + 1.9 °C	+1.9 °C to + 3.9 °C
Precipitations	−6% to −9%	−9% to −18%
Climate extremes	Longer duration of heat waves + high frequency of droughts and floods	

**Table 5:** Main climate impacts on the sheep production systems’ components.

Production System Component	Forage System	Feeding System	Herd and Reproductive Management	Livestock Housing
Climate impacts	Decrease in forage production	Lack of feed resources	Decreased animal/flock performances	Increased thermal stress
	Deterioration of forage quality	Increased feeding costs	Increased abortion and diseases and mortality rates (lambs)	Decreased animal welfare
	Decrease in the surface and production of the grazed area (rangelands and meadows)	Increased pressure on grazing resources	Reduced fertility and prolificacy rates	

## Data Availability

The data presented in this study are available on request from the corresponding author. The data are not publicly available as it is part of the ADAPT-Herd project consortium.
